# Responses of Peripheral Blood Flow to Acute Hypoxia and Hyperoxia as Measured by Optical Microangiography

**DOI:** 10.1371/journal.pone.0026802

**Published:** 2011-10-25

**Authors:** Yali Jia, Peng Li, Suzan Dziennis, Ruikang K. Wang

**Affiliations:** Department of Bioengineering, University of Washington, Seattle, Washington, United States of America; University of Queensland, Australia

## Abstract

Oxygen availability is regarded as a critical factor to metabolically regulate systemic blood flow. There is a debate as to how peripheral blood flow (PBF) is affected and modulated during hypoxia and hyperoxia; however *in vivo* evaluating of functional PBF under oxygen-related physiological perturbation remains challenging. Microscopic observation, the current frequently used imaging modality for PBF characterization often involves the use of exogenous contrast agents, which would inevitably perturb the intrinsic physiologic responses of microcirculation being investigated. In this paper, optical micro-angiography (OMAG) was employed that uses intrinsic optical scattering signals backscattered from blood flows for imaging PBF in skeletal muscle challenged by the alteration of oxygen concentration. By utilizing optical reflectance signals, we demonstrated that OMAG is able to show the response of hemodynamic activities upon acute hypoxia and hyperoxia, including the modulation of macrovascular caliber, microvascular density, and flux regulation within different sized vessels within skeletal muscle in mice *in vivo*. Our results suggest that OMAG is a promising tool for *in vivo* monitoring of functional macro- or micro-vascular responses within peripheral vascular beds.

## Introduction

Blood vessels in either macro- or micro-circulation respond to internal and external stimulations, including tissue metabolites and inspired oxygen concentrations, respectively, which enables blood flow to be regulated according to tissue needs [1], [2]. The availability of oxygen is considered an important factor in metabolic regulation of blood flow, although the mechanism of its action is not totally clear [3]. In brain, the general reactions of the cerebral blood flow (CBF) to different oxygen tensions have been extensively described by numerous investigators, which has been reviewed by [4]. Hypoxia induces hyperemic responses whereas hyperoxia leads to anemic responses of CBF [5]. With respect to alterations in the peripheral blood flow (PBF) in response to different oxygen tension, the results are less understood [3]. Perfusion of the isolated vascular bed of skeletal muscle with hypoxic/hyperoxic oxygen uniformly produces vasodilatation/vasoconstriction related to the severity of the hypoxia/hyperoxia [6]. However, in whole animal studies, where reflex neuro-humoral mechanisms are present, a variety of responses have been observed during arterial hypoxia/hyperoxia [7]. Under the same experimental conditions, some reports indicate an increase [8], [9], [10], whereas others demonstrate a decrease in blood flow to skeletal muscle [6], [11].

Macrovascular responses to inspired oxygen are usually directly measured by the venous occlusion plethysmographic method [7] or estimated by local thermodilution [6], yielding inconsistent results. Likewise, the results from some studies measuring microvascular responses by intravital microscopy [12], [13], [14] are variable. The adjustment of macrocirculation to a change in inhaled oxygen may result in the alteration of microcirculation, e.g. capillaries. For an understanding of the effects of the inhalation of gases with low/high oxygen on the whole circulation, a tool which enables the assessment of macro- and micro-circulation simultaneously would be indispensable. By doing so, macrovascular mechanisms of PBF regulation would be validated by the microvascular mechanisms, and vice versa. Furthermore, the relationship between the two during this physiological perturbation would become clearer.

As a novel extension of optical coherence tomography (OCT) technology, optical microangiography (OMAG) [15] is a new imaging modality capable of generating 3D images of blood perfusion distribution within microcirculatory tissue beds. It is a label-free optical method because the imaging contrasts is produced via endogenous light scattering from flowing red blood cells (RBCs) within open vessels. Recent OMAG development has improved the system sensitivity to blood flow as low as ∼4 µm/s that is sufficient to measure the capillary flows within mouse skeletal muscles [16]. In order to evaluate flow velocity, however, it is required to apply the phase-resolved Doppler technique [17] to the OMAG flow signals so that the differential phase values (thus the axial velocity) are extracted [18]. However, the axial velocity information based on mean frequency shift has significant limitations. These limitations include Doppler angle dependence, aliasing, and difficulty in separating the true flow signal in slow-flow state (e.g. microcirculation) from the noise background. In addition, the Doppler frequency shifts caused by macro-circulation (>50 µm in diameter) flow in fast-flow state are often phase-wrapped, which makes it difficult to obtain the true velocity values. In an attempt to overcome these limitations, in this study, we propose to use “Power Doppler” concept [19], a widely used approach in color Doppler ultrasound modality, to analyze the OMAG flow signals. Analogous to power Doppler ultrasound, the flow signals (i.e., optical reflectance) generated by OMAG indeed demonstrate the integrated power of the Doppler signal [15]; this power is related to the number of RBCs flowing across light beam within a unit time [20], which can be referred to as flux (sometimes also termed as blood flow, flow rate or perfusion rate in literatures). As an alternative means of demonstrating peripheral hemodynamics, in this study, ultrahigh sensitive OMAG flow signals (i.e. power signals) are used to track the changes of both macro-vascular and detailed micro-vascular flow and analyze their relationship under physiological challenge associated with oxygen availability.

## Materials and Methods

### Ethics Statement

The experiments were performed on C57BL/6 adult mice of 20–30 g weight. All experimental animal procedures were in compliance with the Federal guidelines for care and handling of small rodents. The laboratory animal protocol for this work was approved by the Animal Care and Use Committee of the University of Washington (Protocol #4262–01).

### Ultrahigh sensitive OMAG system

**Figure 1:**
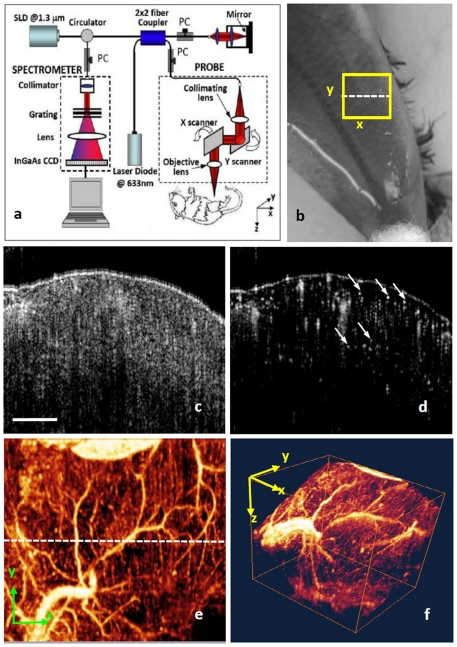
OMAG system and its typical *in vivo* images of detailed PBF within skeletal muscle in mice. (a) is schematic of OMAG system, where SLD is superluminescent diode, PC is polarization controller. (b) shows OMAG imaging area indicated by a yellow box covering an area of the gastrocnemius muscle in mouse hindlimb. (c) is one typical OMAG cross-sectional image (B-scan) of muscular microstructure and (d) is the corresponding blood flow image where some representative capillaries are shown by arrows. 3-D volumetric rendering of blood perfusion within scanned muscle tissue volume are demonstrated by projection view (e) and 3D view (f). In (e), the dash line indicates the corresponding position of chosen B-scan (c/d). White bar is 500 µm, which can apply to (c-e).

The system (Fig. 1a) used to monitor peripheral hemodynamics is similar to that used in our previous work [21], [22]. Briefly, a superluminescent diode with a central wavelength of 1310 nm and a bandwidth of 65 nm was used as the light source, providing a ∼8.9 µm axial resolution in the tissue. In the sample arm, a 30 mm focal length objective lens was used to achieve ∼9 µm lateral resolution. The output light from the interferometer was sent to a spectrometer with spectral resolution of ∼0.141 nm, producing a total depth range of ∼2.2 mm in the tissue. To achieve ultrahigh sensitive imaging of the flow, the spectrometer employed a high speed InGaAs camera (SU1024LDH2, Goodrich Ltd. USA) capable of 92,000 lines per second. We adopted the scanning protocol described in previous work [23], that enabled an imaging rate of 280 frames per second (fps). With this setup, it took ∼3 seconds for the system to acquire a whole 3-D volume dataset, covering an area of ∼2.5 mm in X direction (fast scan) and ∼2.5 mm in Y direction (slow scan) over the sample. With 280 fps imaging rate afforded by the fast camera, the temporal resolution (in terms of cross sectional scan) is sufficient to monitor the time-dependent changes of blood flow in this study.

### Physiological perturbation associated with oxygen

The animal was ventilated with gas using a breathing mask at a gas flow rate of ∼1 L/min. To challenge the animal, we adjusted the O_2_ concentration by balancing N_2_ in the inhaled gas through a gas-proportioning meter (GMR2, Aalborg). A 20% O_2_ gas was initially given to the animal (i.e., normoxia state) and then switched to 10% O_2_ for a period of 5 minutes so that the animal was experiencing acute hypoxia. This was followed by supplying of 20% O_2_ gas for 3 minutes for the animal to recover (back to the normoxia state). And then the gas supply was switched to 100% O_2_ for 5 minutes (i.e, hyperoxia) and finally switched back to 20% O_2_ gas.

### In vivo imaging

Before OMAG imaging, the right hind-limb of the mouse was shaved and depilated. In order to avoid signal attenuation caused by skin and improve the imaging quality, a 4 mm longitudinal skin incision was made to expose our imaging site, the gastrocnemius muscle (see Fig. 1b). The imaging area was kept moist under a piece of plastic foil. During the imaging, the animal was immobilized in a custom-made stereotaxic stage and was lightly anesthetized with isoflurane. The body temperature was kept at 37°C by use of a warming blanket; the body temperature was monitored by a rectal thermal probe throughout the experiment; and the mean arterial blood pressure (MABP) were monitored by CODA monitor (Kent Scientific Corp., Torrington, CT). At initial stage of normoxia, we used OMAG to capture a 3D dataset covering an area of 2.5 mm (X) by 2.5 mm (Y) over the gastrocnemius muscle. During gas challenging periods, OMAG was set continuously acquiring the B-scan images (M-B-scan mode) at a representative position.

### Signal analysis

To provide hemodynamic analysis, we estimated the blood flow (total flux) by integrating the reflectance signals from all vessels across the entire B-scan cross-sectional area, and evaluated its change over time by normalizing the signals with the mean value of baseline. We calculated the microvessel density as the percentage of the number of image pixels with values greater than zero divided by the total pixel numbers for a capillary apparent region. According to the relationship between the density, the mean flux and the total flux within the region, the relative mean flux changes can be obtained by dividing the relative total flux values by the microvessel density values. The relative values obtained at each time point are displayed by the average with standard deviation (SD) calculated from 1000 repeated B-scans.

## Results and Discussion

### Imaging of PBF in skeletal muscle

In Fig. 1, the OMAG *in vivo* imaging results produced by one typical volume dataset [2.5×2.5×2.2 (x-y-z) mm^3^] are shown. Fig. 1c shows one typical cross-sectional image within the OMAG structural volume, which is identical to the conventional OCT image. Figure 1d gives the corresponding blood flow image obtained from the OMAG algorithm [23], where the capillary flows within the cross section of skeletal muscle are abundant from the surface to a depth of ∼1.0 mm, as illustrated by arrows. Rendered with 3D visualization software Amira 4.1.1 (Visage Imaging, Inc.), muscular blood flow distribution (x-y view) and 3D volumetric perfusion image of microvasculature were shown in Fig. 1e and 1f, respectively. Single capillary vessel can be resolved clearly from the dense capillary bundles, which aligned along fiber bundles in longitudinal direction. The transversal cross-connections between capillary bundles are depicted by some arterioles or venules. Aside from the microcirculation, the macrovascular blood flow can also be obtained as shown in Fig. 1e and 1f.

Unlike the previous optical methods, such as laser Doppler imaging and laser speckle contrast imaging which also utilize signals backscattered from flowing particles, this method has the advantage of super high flow sensitivity (down to ∼4 µm/s), making it suitable for detecting the slow blood flows in microvessels, especially capillaries. With its depth-resolved attributes, ultrahigh sensitive OMAG could produce volumetric images in which the microvessel networks within different layers can be easily segmented or visualized [16].

### PBF responses during physiological perturbation

**Figure 2:**
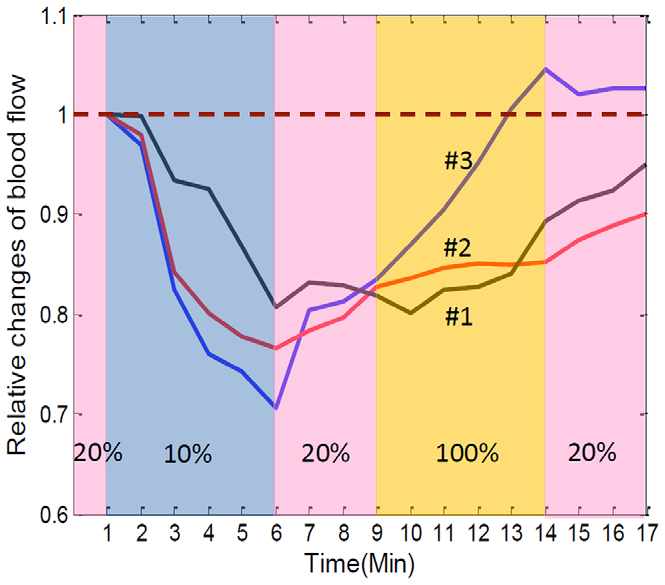
Time lapsed plots showing relative changes of PBF under controlled oxygen concentration, 10% (hypoxia), 20% (normoxia), 100% (hyperoxia), obtained from three individual animals. The relative values were obtained by normalizing the signals with the corresponding baseline values at each time point.

The representative time courses of relative PBF changes from three individual animals are given in Fig. 2. Although their extreme points (maximum and minimum) varied, in general, the responses corresponded well to the temporal locations. When the gas mixture was switched to an acute hypoxia state, a transient blood flow drop occurred within one minute, and continued to 70–82% of respective baseline by 5 min at which time the content of gas was changed to normoxia. Normoxia was sustained for three minutes to allow acute circulatory adjustments to take place. Unfortunately, we observed a partial, rather than a complete restoration of blood flow. When acute hyperoxia was performed, robust augments were shown, but the increasing extents varied in different animals. In animal #3, the PBF rise exceeded the baseline, and achieved the maximum at the end of hyperoxia. During the final stage of three-minute normoxia, the trends of all PBF moved towards baseline wherever they were located.

To explain this dynamics, the MABP variation caused by physiological challenge should be considered. Previous reports indicate that MABP and heart rate are significantly reduced/increased when the mammals are exposed to hypoxia/hyperoxia [5], [7]. The MABP data collected during experiments (Table 1) also shows the influence of oxygen levels on this systemic circulatory parameter. Apparently, the rapid changes in MABP during acute hypoxia/hyperoxia were associated with pressure-passive microvascular PBF. During the hypoxic or hyperoxic 5-min time window, we did not observe any flux adaption on the muscle tissue we imaged, but we cannot exclude the possibility that an adaptation may likely occur during the following chronic phase. For *in vivo* measurements in the whole animal, the external stimulus (low/high concentration of inhaled oxygen) challenges the central nervous system, i.e. the brain, more than distal system, i.e. the muscle in the limb [24]. When a drop in oxygen is detected in brain, the CBF is disturbed and has the priority to be protected over PBF. Specifically, PBF demonstrated a more severe reduction in relative changes of blood flow in response to acute hypoxia-induced drop in MABP compared with those of the CBF (data not shown) and did not display any adjustment during this acute hypoxia stage, indicating muscle is more tolerant than brain tissue to the shortage of oxygen. However, because peripheral tissue perfusion is also essential to the survival of the local tissues and acts to increase blood flow [25], [26], [27], the reduction in blood flux will likely be restored to baseline had we sustained normoxia for a longer time. Here, we were not aiming at monitoring chronic response of microcirculation, so three minutes normoxia was allowed for acute circulatory adjustments to take place, which did not permit PBF be fully restored to baseline. Similar to the PBF response occurring with hypoxia, PBF was elevated due to the increased MABP induced by hyperoxia. However, the PBF changes were much smaller than we observed during hypoxia. Changes in blood flow are related with many factors of physiological condition. For example, under this experimental design, when the controlled oxygen condition was changed to hypoxia, the spontaneous breathing rate may have increased to try to compensate for the lack of oxygen. Although we did not measure breath rate in our studies, increased/decreased breath rate would alter the inhalation of isoflurane and may have additionally resulted in decreased/increased MABP. It is difficult to separate out the reciprocal effects of oxygen challenge and compensatory increase in breath rate when performing these physiological studies for optical imaging. In order to maintain a constant breath rate, artificial ventilation would be required, which is beyond the scope of our study.

**Table 1:** Mean arterial blood pressure (MABP) measured under three physiological states.

	Normoxia	Hypoxia	Hyperoxia
**MABP (mmHg)**	90±4	86±6	96±6

### PBF responses characterized by different vessels

**Figure 3:**
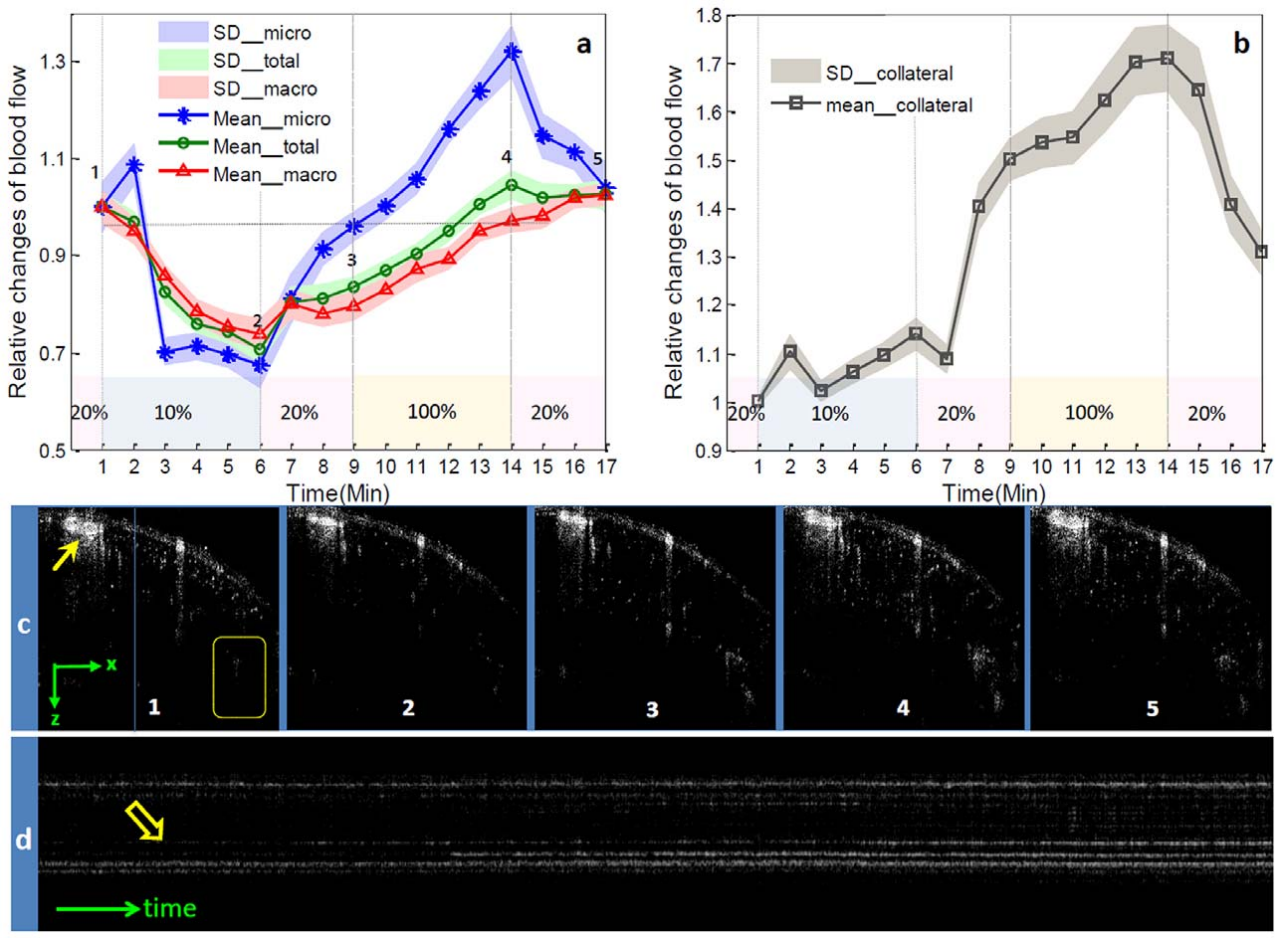
Different types of PBF in response to switching the physiological conditions between hypoxia, normoxia and hyperoxia. (a) shows diverse response of macro-circulation (>50 µm), micro-circulation(<50 µm) and all circulation in the cross-sectional area OMAG scanned. (b) shows the dynamics of collateral vessels located in the region denoted by a box in (c). Each value in (a) & (b) is calculated by normalizing the signals with the baseline at each time point and displayed by mean ± SD. (c) are B-scan images at five typical stages corresponding to the time points labeled by numbers in (a). (d) is one A-scan (Z-direction) over hyperoxic period showing the single vessel dynamics. The position of this A-scan was marked in (c) as the vertical line.

In order to acquire more information with respect to how the macro- and micro-circulation is altered respectively under conditions of regulated PBF, we separated macro- and micro-circulation depending on whether their calibers were over 50 µm and compared their responses to gas variation shown in Fig. 3a. The relative values obtained from a typical experiment at each time point are expressed by average with standard deviation calculated from 1000 repeated B-scans. The observed effect on macro-circulation was much smaller than that in micro-circulation, indicating the varied sensitivity to this potent stimulus within muscular vessels at different flow state. In view of these findings, the action of hypoxia or hyperoxia on macro- and micro-circulation could be virtually distinguished on the B-scan images (shown in Fig. 3c) at five typical stages corresponding to the time points labeled by numbers in Fig. 3a. We visualized the apparent vasoconstriction occurring on the macro-vessels pointed by an arrow in Fig. 3c during systemic hypoxia (1→2) and vasodilation during hyperoxia (3→4) at the macro-circulation level. As a consequence of vasoconstriction/vasodilation, the localized reduction/increase of PBF was found during hypoxia/hyperoxia. On the systemic level, vasoconstriction/vasodilation is one mechanism by which the body regulates and maintains MABP, and, in general, vasoconstriction/vasodilation usually results in an increase/decrease in systemic blood pressure. Therefore, the vasoconstriction/vasodilation appearing under this physiological stress is aimed at dampening or even eliminating the changes of MABP related to oxygen availability. Our finding demonstrated the contribution of PBF on physiological adaption of systemic MABP. Additionally, this initial vasoconstriction in muscle is consistent with a previous report in which the circulation of skeletal muscle was examined by local thermodilution in rabbit [6]. In that report, this early vasoconstriction during hypoxia has been proved to be mediated mainly through sympathetic vasoconstrictor nerves as a result of strong chemoreceptor stimulation [6]. On the micro-circulation level, we found the functional dismissal/recruitment of blood flow through perfused capillaries as indicated by the decreased/increased capillary flow signals which may have contributed to this decreased/enhanced microcirculatory perfusion during transient oxygen-dependent MABP decrease/increase. It's interesting to note that the recruitment of collateral blood flow [28], [29] in macro or micro circulation was initialized at some point during oxygen perturbation. In the region denoted by a box in Fig. 3c, we acquired the dynamics of the collateral circulation; and we are impressed by the protective effect of recruitable collateral vessels in response to increased MABP along with increased inhaled oxygen.

In addition to the M-B-scan mode (repeated cross-sectional scan) which clearly showed the microcirculatory perfusion change, the M-A-scan (repeated axial scan at one spatial location) across some individual capillaries was also able to longitudinally detect the dynamic response of blood perfusion during physiological perturbation. For example, Fig. 3d shows that reflectance signals along one A-line (Z direction) across different vessels changed gradually with time during hyperoxia as more signals denote more blood cells passing through those individual vessels. Specifically, we visualized the whole vascular dynamics in a capillary indicated by an open arrow in Fig. 3d. The capillary was partially opened with some plasma gap from a closed state in the beginning of the hyperoxic stimulus. As the stimulus continued we observed an appearing continuous and bright RBC trace suggesting more blood flux through the capillary during the hyperoxic period. Therefore, in addition to the adaptation of the macro-circulation to changes in the systemic MABP induced by gas challenge, our data indicate micro-circulation is also modulated to expedite the systemic adjustment of blood perfusion in the muscle. It should be emphasized that although we observed the new capillary appearance, we could not find any capillary dilatation due to the accuracy of the capillary diameter evaluation in the current system (∼10 µm).

### PBF response characterized by different parameters

**Figure 4:**
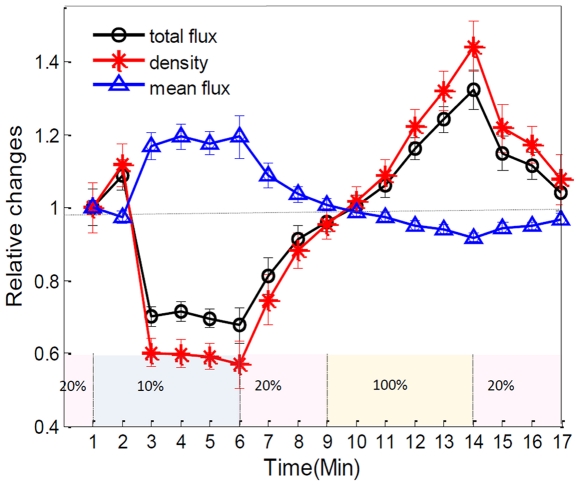
Different flow parameters (total flux, density and mean flux) within microcirculatory PBF in response to physiological challenges. These relative changes (mean ± SD) indicate that the microvessel density change primarily contributes to the total flux change within this microvascular tissue beds while the microvascular mean flux contributes to a minor degree.

Theoretically, an increase/decrease in the total perfusion of the peripheral microvascular network may not only be associated with an increase/decrease in the functional microvessel density, but also related to an increase/decrease in blood flow in each single microvessel; however, little information is available as to which mechanism (either microvessel density or single microvessel flux) plays dominant role in the regulation of regional microvascular PBF. To investigate whether OMAG can shed some light on this, we estimated the functional microvessel density from the serial B-scan images, as described in the “Materials and Methods” section. According to the relationship among the density, mean flux and total flux within the region, the relative mean flux changes can be obtained by dividing the relative total flux values by the microvessel density values. The resulting curves are shown in Fig. 4. Our results show that the perfused microvessel density or volume change mainly accounts for the total blood flux change, whereas, the mean flux of blood flow through individual open microvessels produced little opposite change, which appears to play a secondary role as a mechanism in the regulation of microvascular blood flow to the skeletal muscle during oxygen disturbances. It should be noted that the relative change of mean flux in microvascular PBF correlates well with prior observations based on microscopic observation in which muscle dissection was required [3].

In summary, we presented a preliminary study on using ultrahigh sensitive OMAG to monitor PBF changes during hypoxia and hyperoxia in mice. By analyzing OMAG reflectance signals from moving blood cells, we have shown that OMAG is sensitive enough to distinguish changes in hemodynamics within either macro- or micro-circulation, and has a potential to differentiate the hemodynamics produced by macro-circulation from that by micro-circulations. OMAG was able to describe the vascular diameter change on macrovascular level and demonstrate the microvascular distribution. More interestingly, OMAG could provide evidence that the hemodynamics occurred in single capillaries during systemic oxygen challenge. Furthermore, OMAG could elucidate the pattern of microhemodynamic changes with respect to different flow parameters, such as, density, mean and total flux. Our data provide proof of concept that ultrahigh sensitive OMAG is a highly promising technique for use in the pre-clinical and clinical settings to determine changes in macro- and micro- hemodynamics under physiological and pathophysiological conditions.
